# Effects of different doses of tamoxifen on the sperm parameters and chromatin quality in mice: An experimental model

**DOI:** 10.18502/ijrm.v17i4.4553

**Published:** 2019-06-13

**Authors:** Sepideh Sadeghi, Ali Reza Talebi, Abbas Shahedi, Mohammad Reza Moein2 MD., Abolghasem Abbasi-sarcheshmeh

**Affiliations:** ^1^ Department of Biology and Anatomical Sciences, Shahid Sadoughi University of Medical Sciences, Yazd, Iran.; ^2^ Research and Clinical Center for Infertility, Yazd Reproductive Sciences Institute, Shahid Sadoughi University of Medical Sciences, Yazd, Iran.

**Keywords:** *Tamoxifen*, * Sperm*, * Chromatin*, * Mice*

## Abstract

**Background:**

Tamoxifen (TX) is widely used for the treatment of male factor and idiopathic infertility. It has been shown that TX induces sperm production and so improves male fertility.

**Objective:**

This study evaluated the effects of different doses of TX on the sperm parameters and chromatin quality in mice.

**Materials and Methods:**

In this research, 24 male NMRI mice were divided into three groups including group A: control animal receiving vehicle; group B: the group receiving basal diet and TX 0.4 mg/kg/day; and group C: the group receiving basal diet and TX 0.6 mg/kg/day for 35 days. Thereafter, epididymal spermatozoa were analyzed for standard parameters and nuclear chromatin quality using Aniline Blue (AB) and Toluidine Blue (TB) staining.

**Results:**

The results indicated that although the TX did not affect the sperm count, motility, and viability parameters, it could elevate the percentage of sperm cells with abnormal morphology and abnormal chromatin at both doses. In addition, in comparison with the control mice, a significant elevation was observed in spermatozoa with residual histones (assessed by AB staining) at high doses of TX.

**Conclusion:**

Our experimental data in mice suggested that the use of TX for treating male infertility might increase the rates of spermatozoa with abnormal chromatin in a dose-dependent manner.

## 1. Introduction

Male and female infertility is a common problem with 10–15% of couples being infertile, 50% of which are male factor (1). On the other hand, more than 30% of male infertility is unexplained (2) and about 10% of these men have azoospermia. Tamoxifen (TX) lies in selective anti-estrogen receptor modulators (SERM) drug category. It has been used in the treatment of infertile men with idiopathic infertility, oligospermia, and nonobstructive azoospermia (3). Daily use of 20 mg TX in men with sexual dysfunction for six consecutive months increased the sperm count in the ejaculate (4). One study indicated that anti-estrogen drugs such as TX could improve the results of spermatogenesis in testicular samples in men with non-obstructive ozoospermia where TX was used for three consecutive months. In addition, the use of this drug has a positive effect on patients with sperm maturation, so taking TX can increase the probability of pregnancy through microinjection (3). Today, the detection of sperm nuclear chromatin integrity is a very important issue in male fertility assessments (5). The sperm chromatin is highly structured that contains DNA and heterogeneous nucleoproteins. The dense and insoluble nature of sperm chromatin protects the genetic material of the cell while passing through the male and female genital tract (6). The results of another study suggested that in varicocele patients, there is a high rate of spermatozoa with abnormal chromatin condensation, which is one of the possible causes of male infertility due to varicocele (7).

Our present study aims to investigate the possible effects of TX on sperm parameters and chromatin condensation. Although TX is now used as a therapeutic drug for infertile men to increase sperm count, we investigated the effect of TX on the sperm chromatin quality and its condensation, which is a new issue in male reproductive evaluations. On the other hand, we proposed the safe dosage of TX to reduce the damage of spermatozoa caused by this drug. Therefore, the different doses and the chromatin assessments are the novelties of the present study.

## 2. Materials and Methods

### Animals

In this experimental study, 24 adult male NMRI mice weighing 30–35 gr were divided into three groups including two experimental (B: dose 0.4 mg/kg/day and C: dose 0.6 mg/kg/day) and one control (C) groups, each containing eight mice. The animals of group B received basal diet and TX (low dose) orally, group C received basal diet and TX (high dose:), and group A as the control animals received basal diet without any treatments for 35 days. All mice were maintained at a temperature of 22–23°C, the humidity of 50–55%, and a light/dark cycle of 14/10 hr.

### Tamoxifen (TX) treatment

The animals in the two experimental groups B and C were orally administrated 0.4 mg/kg/day and 0.6 mg/kg/day of TX (Iran Hormone Co.), respectively. To prepare the drug dosages, the 10 mg pills of TX were suspended uniformly in water by sonication and then administrated daily using a mice-feeding tube.

### Epididymal sperm aspiration and sperm analysis

At the end of day 35, the mice were killed through cervical dislocation and the tail of right epididymis of each mouse was intercept and placed in 1 ml of pre-warmed Ham's F10 medium. The dishes were placed in the incubator (37°C) for 15 min. Swim-out spermatozoa were used to calculate and determine the count, motility, morphology, and viability. Motility was determined in three categories including progressive (fast and slow), non-progressive, and immotile spermatozoa. Sperm morphology was assessed by Papanicolaou staining and the percentage of sperm cells with normal morphology was determined. For viability, we used Eosin test where 200 spermatozoa were counted (8, 9).

### Sperm chromatin assays 

For evaluating the sperm chromatin condensation, we used cytochemical methods including Aniline Blue (AB) and Toluidine Blue (TB) stainings (7). All dyes and chemicals were purchased from Sigma Aldrich Company (St Louis, MO, USA).

#### Aniline blue (AB) staining

Nuclear histones with a large number of basic amino acids like Lysine react with acidic dyes such as AB. Spermatozoa with blue-stained nuclei are considered as immature spermatozoa (AB+) which have excessive histones, while colorless spermatozoa are considered as normal or mature spermatozoa (AB-). For this staining, air-dried smears were fixed in 3% buffered glutaraldehyde in phosphate buffer 0.2 M (pH = 7.2) for 30 min at room temperature, and then they were stained with aqueous AB 5% stain in acetic acid 4% (pH = 3.5) for 7 min. In each slide, 200 sperm cells were counted and the percentages of AB+ and AB- were determined (10).

#### Toluidine blue (TB) staining

Toluidine blue is a metachromatic color, which establishes both chromatin condensation and DNA fragmentation via connection to phosphate groups of DNA molecules (8). To do this staining, air-dried smears were fixed in fresh ethanol-acetone 96% (1:1) at 4°C for 30 min, and then hydrolyzed in HCl 0.1 N at 4°C for 5 min. Next, the sperm smears were washed three times in distilled water for 2 min, subsequently, TB staining were done 0.05% in citrate phosphate 50% for 10 min at room temperature. In each slide, 200 spermatozoa were counted under light microscopy using ×100 eyepiece magnifications (7, 10). In TB staining, the sperm cells were scored by the color of heads with score 0: light blue (good chromatin); 1: dark blue (mild abnormal chromatin); 2: violet; and 3: purple (severe chromatin abnormality). So, scores 1, 2, and 3 represent TB+ or sperm cells with abnormal chromatin.

### Ethical consideration

Animal usage and the protocols were approved by the Institutional Animal Care and Ethics Committee of Biological Sciences of Yazd University (IR.SSU.MEDICINE.REC.1395.9).

### Statistical analysis

The results were introduced into SPSS software (Statistical Package for the Social Sciences, version 18.0, SPSS Inc., Chicago, Illinois, USA). The statistical analysis was performed to compare the groups by one-way ANOVA and compare between groups by the post hoc-Tukey analysis. In all analyses, p < 0.05 was considered as statistically significant.

## 3. Results

**Figure 1:**
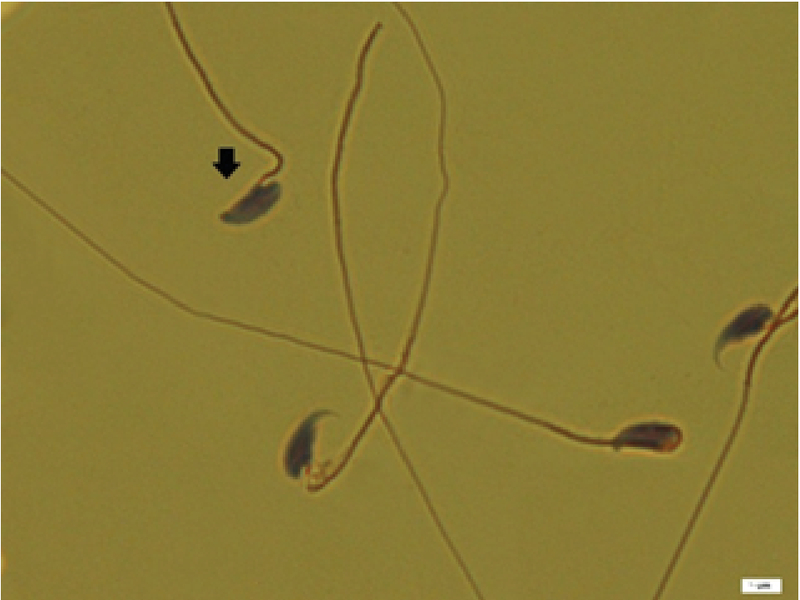
Morphological abnormalities of sperrms; the arrow indicates abnormal spermatozoon. Papanicolaou staining (magnification 100×).

**Figure 2:**
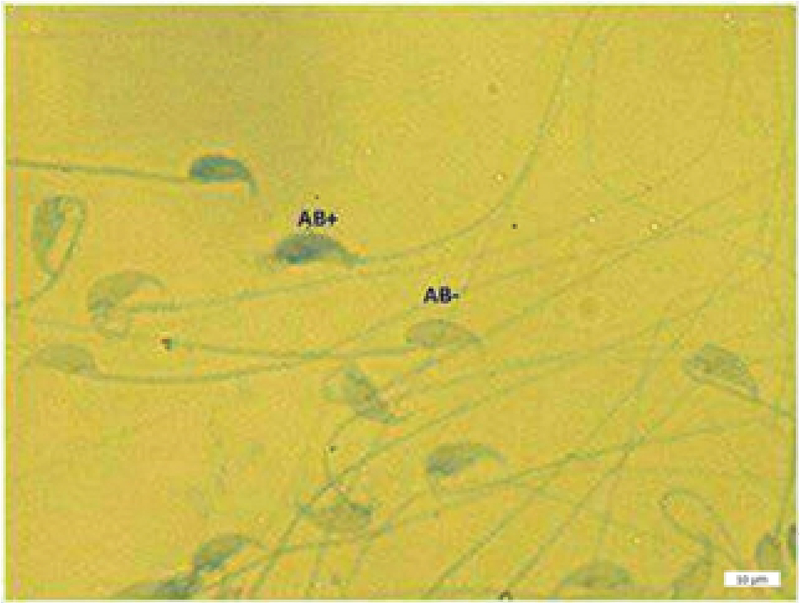
Two Aniline blue-reacted sperms (AB+) and one normal sperm cell (AB-); Aniline blue staining (magnification 100×).

**Figure 3:**
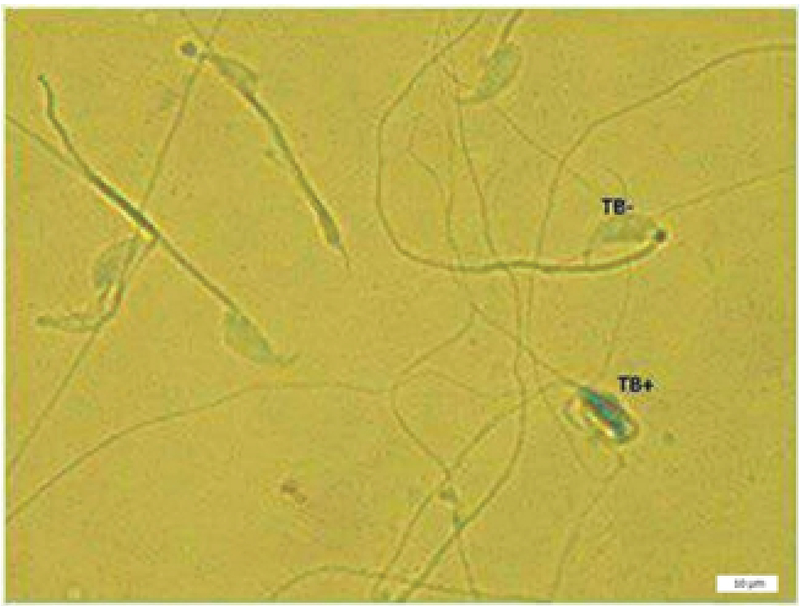
Toluidine blue staining of sperms; TB+ indicates sperm cells with abnormal chromatin and TB- indicates sperm cells with normal chromatin (magnification 100×).

Considering the effects of TX on sperm counts, viability, and motility, no significant differences were observed between the groups, but it can increase the percentage of sperm cells with abnormal morphology. Indeed, the percentage of sperm with an abnormal morphology was higher in groups B and C than in group A (Figure 1). There was no significant difference between B and C groups. However, in the chromatin study, the AB staining showed a statistically significant difference only at a high dose (group C) (Figure 2). In addition, concerning TB staining, there was a significant difference between both doses B and C with group A (Figure 3), but there was no significant difference between groups B and C (Table I).

**Table 1:** The results of sperm analysis in the two experimental groups: dose 0.6 mg/kg/day (C), dose 0/4 mg/kg/day (B), and control group (A)


**Group**	**Group A**	**Group B (0/4mg/kg/day)**	**Group C (0/6mg/kg/day)**	**p-value**
Count	42.7 ± 4.6	43.8 ± 6.3	43.2 ± 5.3	0.919
Fast, slow motility (%) (grade a-b)	60.2 ± 5.3	60.6 ± 4.9	61.8 ± 3.9	0.778
Non progressive motility (%) (grade c)	13.0 ± 5.07	13.7 ± 4.2	17.1 ± 7.5	0.336
Immotile motility (%) (grade d)	25.6 ± 4.5	25.6 ± 6.1	21.0 ± 9.0	0.317
Viability (%)	62.8 ± 2.9	61.7 ± 12.6	64.8 ± 3.5	0.721
Normal morphology (%)	68.5 ± 12.6	54.7 ± 6.0*	54.1 ± 7.7*	0.008
AB	16.0 ± 4.9	19.1 ± 1.5	25.7 ± 4.9#	0.000
TB	6.7 ± 1.4	12.6 ± 5.7*	13.1 ± 1.1*	0.002
white<bcol>5</ecol>Note: The results are shown as mean ± SD, p < 0.05. For data analysis, we used standard one-way ANOVA test
white<bcol>5</ecol>Parameters and AB: Aniline blue, TB: Toluidine blue
white<bcol>5</ecol>“*”Significant difference between the control group and the two experimental groups considering the morphology sperm and TB staining
white<bcol>5</ecol>“#”Significantly different between the control group and group C on AB staining

## 4. Discussion

The findings of the present study suggested that taking different doses of TX does not affect the parameter, but it weakens sperm morphology and quality of chromatin. It has been found that TX may increase sperm count and today it is used as a good therapeutic way in the treatment of male-factor infertility. There are several clinical and experimental studies indicating the effects of TX on non-obstructive azoospermia (3, 4). Although there are many data on the relationship between TX and sperm parameters, our report may be considered as a novel work representing the side effects of TX on chromatin sperm. In one study conducted on the beneficial effects of TX on sperm improvement in infertile men with non-obstructive azoospermia, the elevated sperm count was observed in the azoospermia and maturation syndrome groups (3). The use of TX enhances the thickness of the tunica albuginea and shrinks the diameter and thickness of the seminiferous tubules. There was also a significant reduction in the number of spermatogonial cells including spermatogonia types A and B, primary spermatocytes, spermatoids, spermatozoa, Sertoli, and Leydig cells (11). Another study in 2005 revealed that TX did not have any effect on sperm count at a dose of 0.4 mg/kg/day for 60 days (12). Similarly, in our study, the use of this drug had no effect on the number of epididymal sperm at a dose of 0.4 and 0.6 mg/kg/day for 35 days. In contrast, a study in 2007 showed that the treatment of patients with the dose of 0.4 mg/kg/day TX significantly reduced the sperm concentration and motility in ejaculated and epididymal spermatozoa; but it did not affect sperm survival rates (13). In our study, TX had a negative effect on sperm chromatin quality, which can lead to negative effects on fertility. Another experimental study in 2004 indicated that TX citrate has a negative effect on the testicular and prostatic size and decreases the level of testosterone in dogs (14). Recent studies have shown that the use of TX drug on sperm structure and oxidative stress levels in oligoasthenoteratospermic patients can significantly improve malondialdehyde, sperm concentration, sperm morphology, pathology, and mitochondrial abnormalities (15). The use of estrogen antagonist drugs such as TX can improve infertility in sperm count in infertile patients with less side effects (16). On the other hand, our results determined some anomalies in sperm chromatin, which are observed following TX administration. It has been found that high percentages of sperm protamine defects could be an important cause for idiopathic abortion (17).

A study in 2009 suggested that another side effect of TX therapy may be a significant reduction of DNA methylation (IGF2-H19ICR) in region-specific DNA methylation in rat spermatozoa, but overall, the total methylation level is not altered. The spermatozoa DNA methylation (IGF2-H19ICR) of specific DNA methylation in rat spermatozoa may affect fetal viability after implantation (18). A study performed in 2002 demonstrated that TX treatment could cause pre-implantation embryo loss at the 2-4-cell stage and post-implantation embryo loss at around days 8-9 of gestation. One of the possible mechanism of the effects of TX on the fetus is reducing androgens and blocking estrogen receptors (19). In addition, another study suggested that TX citrate reduces sperm chromosomal condensation via reducing the testicular levels of the transition proteins 1, 2 (TP1, TP2), protamine 1 (P1), and cyclic adenosine 3', 5' monophosphate (AMP) response element modulator-τ (CREM) proteins which play an important role in chromatin compaction during spermatogenesis. It is believed that TX citrate has an effect on the expression of these genes at both levels including transcriptional and post-translational levels (12). Our study can open a new avenue for broader research to determine the positive and negative effects of TX drug use on the quality of chromatin. Our study limitations include the problems related to mice death and small sample size.

## 5. Conclusion

Our results suggested that the use of TX at different doses could not affect the sperm parameters (count-viability-motility) in one duration of spermatogenesis. In addition, TX increases sperm with abnormal morphology and may diminish the quality of sperm chromatin at both doses by a reduction in chromatin compaction which is very important in male fertility and embryonic development. Therefore, in the therapeutic usage of TX, we should consider the detrimental effects of this drug on the sperm chromatin structure.

##  Conflict of Interest

The authors declare that there is no conflict of interest.
